# Tuberculosis: Pathogenesis, Current Treatment Regimens and New Drug Targets

**DOI:** 10.3390/ijms24065202

**Published:** 2023-03-08

**Authors:** Shahinda S. R. Alsayed, Hendra Gunosewoyo

**Affiliations:** 1 Curtin Medical School, Faculty of Health Sciences, Curtin University, Bentley, Perth, WA 6102, Australia; 2 Curtin Health Innovation Research Institute, Faculty of Health Sciences, Curtin University, Bentley, Perth, WA 6102, Australia

**Keywords:** tuberculosis, TB pathogenesis, latent TB, TB treatment regimens, mycobacterial drug targets, anti-TB drug candidates

## Abstract

*Mycobacterium tuberculosis* (*M. tb*), the causative agent of TB, is a recalcitrant pathogen that is rife around the world, latently infecting approximately a quarter of the worldwide population. The asymptomatic status of the dormant bacteria escalates to the transmissible, active form when the host’s immune system becomes debilitated. The current front-line treatment regimen for drug-sensitive (DS) *M. tb* strains is a 6-month protocol involving four different drugs that requires stringent adherence to avoid relapse and resistance. Poverty, difficulty to access proper treatment, and lack of patient compliance contributed to the emergence of more sinister drug-resistant (DR) strains, which demand a longer duration of treatment with more toxic and more expensive drugs compared to the first-line regimen. Only three new drugs, bedaquiline (BDQ) and the two nitroimidazole derivatives delamanid (DLM) and pretomanid (PMD) were approved in the last decade for treatment of TB—the first anti-TB drugs with novel mode of actions to be introduced to the market in more than 50 years—reflecting the attrition rates in the development and approval of new anti-TB drugs. Herein, we will discuss the *M. tb* pathogenesis, current treatment protocols and challenges to the TB control efforts. This review also aims to highlight several small molecules that have recently been identified as promising preclinical and clinical anti-TB drug candidates that inhibit new protein targets in *M. tb*.

## 1. Introduction

**Figure 1:**
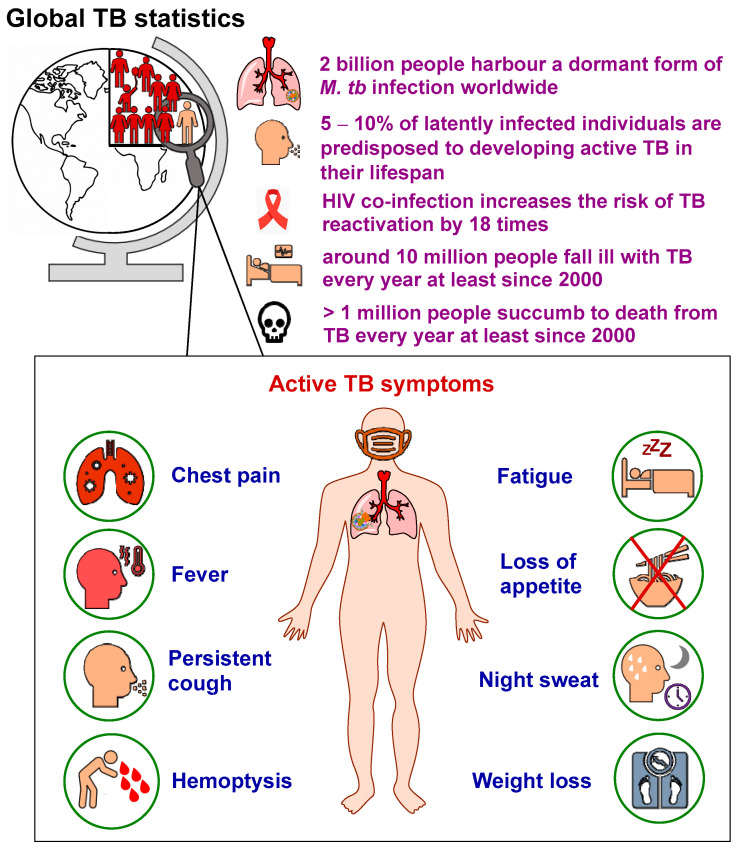
General TB statistics and main symptoms of pulmonary TB.

In 1882, Robert Koch identified the tubercle bacillus, also known as *Mycobacterium tuberculosis* (*M. tb*), as the etiologic agent of tuberculosis (TB) [1]. Since his discovery, the TB epidemic seems to be unabated, spreading in every corner of the globe. TB is a highly contagious airborne disease and one of the top causes of death worldwide [2]. Although the disease typically affects the lungs (referred to as pulmonary TB), it can also spread to other parts of the body (known as extrapulmonary TB) [2]. *M. tb* can stay dormant for years and persist in the body without any indication of illness, in which many people become asymptomatic carriers (inactive TB) [3]. According to the 2022 World Health Organisation (WHO) report [2], around one quarter of the world’s population (2 billion) are latently infected with *M. tb* (Figure 1). In the individuals carrying latent TB infections (LTBI), the estimated lifetime risk for TB reactivation is 5–10% [2]. Indeed, the dormant mycobacteria can be awakened (active TB), particularly in the immunocompromised patients, such as those who are co-infected with the human immunodeficiency virus (HIV). Among people living with HIV (approximately 38 million), the risk of developing TB is estimated to be 18 times higher than people without HIV [2,4]. When the stalemate is broken, TB reactivation occurs and the bacterial burden soars, wherefore the disease becomes symptomatic [5].

Early diagnosis and successful treatment of TB is crucial to prevent further spread of the bacteria and development of resistant strains [6]. Several diagnostic techniques are commonly employed, including immunological, radiographical, microscopical, bacterial culture and clinical methods. Immunological tests, such as QuantiFERON-TB Gold (QFT) and Tuberculin skin test (Mantoux test) are mainly used for the purpose of screening and ruling out TB infection [6]. Similarly, radiography (Chest X-rays) is a screening tool used to diagnose active pulmonary TB; however, it cannot help in detecting latent TB infection. Sputum smear microscopy is a very efficient and widely used tool in TB diagnosis, in which the TB bacteria are stained with Ziehl–Neelsen stain, but low sensitivity and lack of differentiation between *M. tb* and other acid-fast bacilli are key caveats of this method [6]. Unlike smear spectroscopy, sputum culture is a highly specific and sensitive diagnostic TB method wherein Löwenstein–Jensen medium is used to culture the TB bacteria. However, since *M. tb* is a slow-growing organism, it takes at least two weeks (sometimes 6–8 weeks) for the colonies to appear, which further delays the diagnosis and treatment. Finally, in 5–10% of TB-infected individuals, several signs and symptoms develop, which allow for clinical diagnosis [6]. The clinical manifestations of active pulmonary TB may include pleuritic chest pain, low-grade fever, prolonged productive cough, haemoptysis, fatigue, loss of appetite, night sweat and weight loss [6,7] (Figure 1).

Geographically, in 2021, the majority of people who developed TB were located in the WHO South-East Asian region (45%), followed by the WHO African region (23%) and the WHO Western Pacific region (18%). Four countries accounted for more than half of the global TB burden: the two WHO South-East Asian countries, India (28%) and Indonesia (9.2%), in addition to the two WHO Western Pacific countries, China (7.4%) and the Philippines (7.0%) [2]. Although the fraction of people with LTBI predisposed to TB reactivation is seemingly small, roughly 10 million people fall ill with TB annually at least since 2000 [2]. In addition, from 2000 to 2021, approximately 1.4 to more than 2 million people died from TB each year, with the highest mortality rates occurring between 2000 and 2010. The latest 2022 WHO report documented that in 2021 TB claimed the lives of more than one million people worldwide (an estimated 1.4 million and 0.2 million deaths among the HIV-negative and the HIV-positive cohorts, respectively). In fact, until the recent coronavirus 2019 (COVID-19) pandemic, TB-related fatalities surpassed the deaths toll from any other single infectious agent, including HIV/AIDS [2].

Unfortunately, no effective vaccine is currently available to prevent TB disease in adults, either before or after exposure to *M. tb* [2,4]. Nonetheless, the only licenced TB vaccine, bacille Calmette-Guérin (BCG), which was developed nearly a century ago, can confer moderate protection in infants and children, especially from severe forms of TB (miliary TB and TB meningitis). Indeed, while anyone anywhere can get infected with TB, most people (about 90%) who develop active TB are adults, with more incidents among men than women [2,4]. Therefore, there is a pressing need for a more efficacious vaccine that provides immunity against all forms of TB across all age spectrums. In addition, new anti-TB drugs that are superior to the currently available treatment options in terms of efficacy, tolerance and treatment duration are urgently required to cure and curb the spread of TB. In this review article, we will discuss TB pathogenesis, current treatment regimens, challenges to the global TB control, as well as current TB drug targets and their corresponding drug candidates.

## 2. TB Pathogenesis

**Figure 2:**
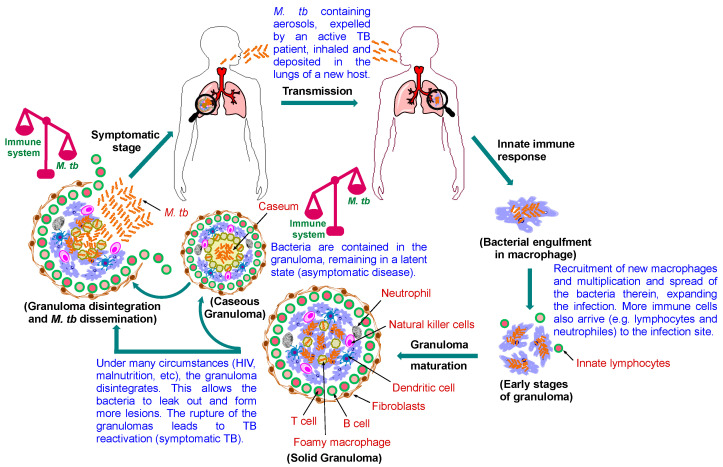
Pathophysiology of pulmonary TB. Following the *M. tb* transmission to the new host, the bacilli enter the lung and get ingested by macrophages. Further immune cells are recruited to wall off the infected macrophages, leading to the formation of the granuloma, the hallmark of TB. Healthy individuals remain latently infected, and the infection is kept at bay at this stage, but it is prone to the risk of reactivation. Foamy macrophages release their lipid content when they necrotise, leading to caseation (cheese-like structure). Caseum is a decay manifested at the core of the granuloma that compromise its rigid integrity. As the granuloma develops, the bacilli commence to seep out of the macrophages into the caseum layer. When the reactivation occurs, *M. tb* proliferates and the bacterial load becomes overwhelmingly high, whereupon the granuloma rupture, disseminating the bacteria to the airways. The bacilli are then expectorated as contagious aerosol droplets, restarting the cycle, infecting other individuals.

The pathogenic life cycle of *M. tb* is illustrated in Figure 2. TB is transmitted via *M. tb*-containing aerosol droplets, propelled by active TB patients when they cough, sneeze or talk [7]. After the new host inhale the TB bacteria, they travel through the respiratory tract and reach the lung. At this point, the host’s innate immune system comes into play to quell the infection, whereupon the tubercle bacilli are internalised by alveolar macrophages. When the macrophages fail to inhibit or destroy the bacilli, the bacteria multiply within their intracellular environment, get released, then phagocytosed by other alveolar macrophages and the cycle continues [7]. Lymphocytes are then recruited to the infection site, initiating a cell-mediated immune response, in which a pile of immune cells arrives, attempting to sequester the bacteria and limit further multiplication [8]. At this stage, the host remains asymptomatic, and the TB bacteria may get eliminated completely or step into latency inside the granuloma [9]. However, in the setting of impaired immunity, the disease immediately progresses into active TB with clinical symptoms [9].

The granuloma is the cardinal feature of pulmonary TB, which is an amorphous collection of macrophages and other immune cells aimed at restricting the bacterial spread [9]. In immunocompetent individuals, although the granuloma is unable to eliminate the pathogen, it restrains the bacilli and halts the progression to the active disease [8]. However, the bacteria still survive, avoiding death by blocking the phagolysosome fusion and subverting the host’s immune response. This process establishes a hospitable niche for *M. tb* where it can survive for decades, outwitting the immune system and persisting in a non-replicating or slowly replicating state [8]. In this case, the patient is still non-infectious and asymptomatic (latently infected). Notably, one of the challenges facing the current TB therapy is targeting this tenacious pathogen inside the granuloma.

As the granuloma matures, macrophages differentiate into foamy macrophages and other various morphotypes (Figure 2) [10]. The centre of the granuloma may necrotise as a result of the necrotic lysis of the host immune cells forming what is referred to as caseum (caseous granuloma) [11]. Indeed, the accumulating soft necrotic debris, located in the core of the granuloma, resembles cheese (earning it the name caseum). Foamy macrophages, which are characterised by accumulated lipid droplets, distribute around the necrotic foci of the granuloma [12]. Importantly, the *M. tb*-induced dysregulation of host lipid metabolism, via disrupting the balance between the influx and efflux of lipid particles from the serum and sequestration thereof, were found to play a critical role in the disease progression [10]. This disturbance in lipid metabolism promotes the formation of foam cells, which support bacterial persistence and eventually result in the accumulation of caseum in the granuloma [10]. In addition, mycolic acids (MAs), which are the major lipid components of the *M. tb* robust cell wall that are essential for the mycobacterial growth and survival, were reported to contribute to the differentiation of macrophages into foam cells [13,14]. The resulting caseous lesions serve as reservoirs, encasing and sheltering the tubercle bacilli, which maintain the dormancy of the bacteria [15]. However, in the late stage of the disease, the caseous core softens and cavitation takes place, leading to resuscitation of the bacteria, and the patient develops active TB, culminating in transmission of the infectious bacilli into a new host (Figure 2) [10,15]. This life-threatening transformation largely relies on the effectiveness of the host’s immune response in limiting the bacterial replication [8].

Even though the main cause of TB reactivation is ascribed to HIV co-infection, other conditions may also switch the quiescent infection to an active one. These triggering factors include malnutrition, immune suppressive medications, chemotherapy, uncontrolled diabetes mellitus, sepsis, drug or alcohol addiction, chronic renal failure, smoking and malignancy [8]. When the host is immunocompromised, the dormant bacilli, originally enclosed in the granuloma, will reactivate and replicate, accompanied by the granuloma liquifying and cavitating [12]. Accordingly, the structure of the granuloma wanes, and the contagious bacteria are released, which results in the formation of cavitary lesions, signifying the lung damage observed in TB patients [8,16]. Furthermore, the caseous material serves as a fertile source of nutrients that promotes the growth of the pathogen to an overwhelming burden [16]. Finally, the bacilli spread throughout the lung and find their ways to the blood capillaries, paving the way not only for transmission to other people, but also for dissemination to other organs [16]. At this stage, the disease becomes highly infectious and symptomatic (active TB). As lung histology during the active disease indicates the coexistence of granulomas at different stages of development, granuloma progression is thought to correlate with TB reactivation [16]. In fact, while three main types of granulomas, namely solid, necrotic and caseous granulomas, have been discerned, they form a continuum and should not be treated as separate entities. Solid granuloma (Figure 2) form at the early stage of the disease and entails tissue damage, hence, histologically, it correlates both the pathology and *M. tb* containment. It is typically enclosed by a fibrotic wall, lacks central necrosis and comprises various immune cells, which are the key mediators in controlling the infection, especially T lymphocytes [16]. Indeed, the *M. tb* burden is low in solid granulomas which make them prevalent in LTBI. As the disease progresses, the centre of the solid granuloma starts to necrotise (necrotic granuloma), paving the way for the revival of the dormant bacteria later in the process when the necrotic centre becomes larger and liquify (caseous granuloma; Figure 2). An in-depth description of granulomas from latent to active TB has been covered elsewhere [16,17,18,19].

## 3. Current Treatment Regimen for Drug-Sensitive (DS) TB

**Figure 3:**
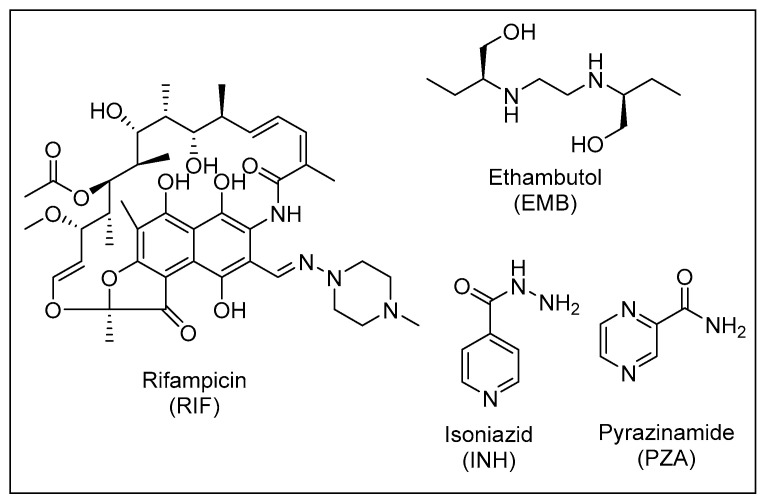
The four front-line anti-TB drugs.

The current recommended treatment for DS-TB involves a combination of four antibiotics: isoniazid (INH), rifampicin (RIF), pyrazinamide (PZA) and ethambutol (EMB), which were all discovered nearly 60 years ago [20] (Figure 3). This four drug cocktail should be administered for at least 6 months under directly observed treatment (DOT) to ensure high rates of treatment success and cure. The treatment involves two phases: the initial phase, which comprises administering the aforementioned four drugs for two months, and the continuation phase treatment with INH and RIF for the last four months to kill the dormant bacteria [20].

The four drugs target *M. tb* via different mechanisms of action. Briefly, INH is a prodrug that upon activation inhibits the enoyl-acyl carrier protein reductase (InhA), which is a key enzyme in the MAs biosynthetic process [21]. MAs are the primary mediators of the hydrophobic attributes and lack of permeability of the mycobacterial outer coating [13]. RIF binds to the ꞵ-subunit of the bacterial RNA polymerase and exerts its bactericidal activity by inhibiting the early steps of gene transcription [22,23]. Like INH, PZA is a prodrug that gets activated after diffusing into the TB granuloma by the pyrazinamidase enzyme to pyrazinoic acid (POA), which subsequently kills the *M. tb* bacillus inside the granuloma [24]. However, the mode of action of PZA is still enigmatic. EMB is a bacteriostatic drug that inhibits the synthesis of arabinogalactan and lipoarabinomannan, two essential components of the mycobacterial cell wall, by targeting the three arabinosyltransferases EmbA, EmbB and EmbC [25].

Despite the effectiveness of the four front-line anti-TB agents against DS-TB, several adverse side effects are associated with this regimen, including liver dysfunction, peripheral neuropathy, erythromelalgia, ocular toxicity, central nervous system (CNS) toxicity, gastrointestinal (GI) intolerance and skin rash [26,27,28]. Poor patient compliance owing to these unwanted side-effects, high pill count and protracted duration of therapy in addition to the overuse/misuse of antibiotics contributed to the emergence of DR *M. tb* strains [26].

## 4. Challenges to the Global Control of TB

### 4.1. Drug-Resistant (DR) TB Crisis

**Figure 4:**
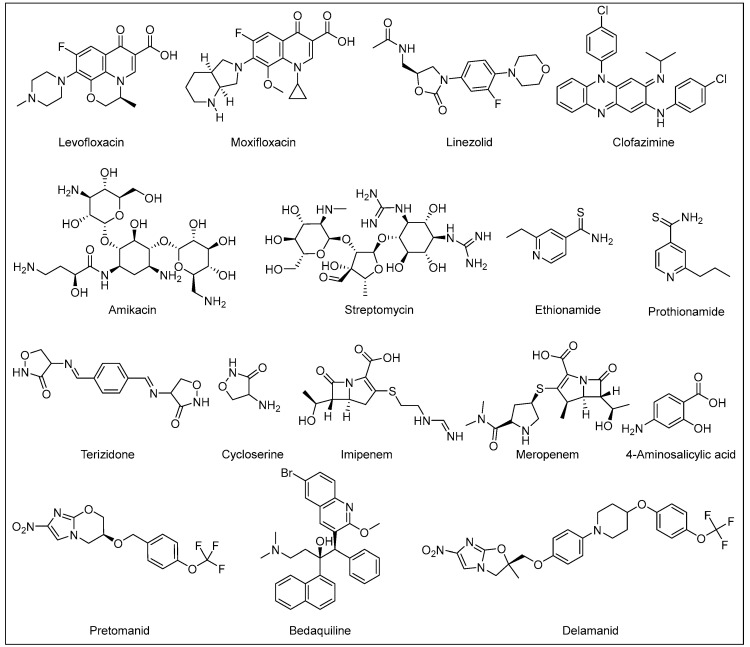
Current second-line anti-TB agents.

The therapeutic approach for DR-TB and the prognosis thereof is significantly correlated to the resistance pattern; however, the clinical management of DR-TB is generally complicated. Multidrug-resistant TB (MDR-TB) is defined as resistance to INH and RIF, the two most powerful front-line anti-TB drugs [2]. In 2021, there were an estimated 450,000 MDR-TB incident cases. The cure rates for MDR-TB are typically significantly lower than DS-TB [2]. The 2019 WHO recommended second-line regimen for MDR-TB (Figure 4) is an 18–20 months treatment protocol, contingent on the patient’s response to therapy. The MDR-TB medication regimen consists of at least four drugs in the intensive phase: three drugs from group A [linezolid, bedaquiline (BDQ) and moxifloxacin/levofloxacin] and one drug from group B (clofazimine, or terizidone/cycloserine) [29]. At least three of these drugs should be prescribed for the rest of the treatment (continuation phase) after BDQ is stopped. Two drugs in group B should be prescribed if only one or two drugs from group A are used [29]. If the *M. tb* strain is resistant to one or more of the preceding drugs, drugs from group C [delamanid (DLM), streptomycin/amikacin, EMB, PZA, 4-aminosalicylic acid, imipenem, meropenem, ethionamide/prothionamide, high dose INH] should be added to the regimen [29,30].

In 2020, the WHO recommended a shorter all-oral regimen for MDR-TB (9–11 months) to make it easier for the patients to complete the therapy in comparison to the aforementioned longer regimen [31]. The initial phase of this shorter treatment protocol comprises administering a cocktail of BDQ, moxifloxacin/levofloxacin, clofazimine, ethionamide/prothionamide, INH (high dose), PZA and EMB for four months (with a possibility of extension for a maximum of six months if the patient’s culture or sputum smear remains positive by the end of the fourth month). Regardless, BDQ should be used for 6 months in total. The continuation phase is fixed at 5 months, entailing the administration of moxifloxacin/levofloxacin, clofazimine, PZA and EMB [31]. It is worth noting that BDQ and DLM, which were recently implemented in the second-line regimen, are the first anti-TB drugs with new mechanisms of action to be approved for treatment of TB in more than half a century; RIF was approved for clinical use in Italy in 1968 and in USA in 1971 [32,33]. BDQ was the first to be approved by the United States Food and Drug Administration (US FDA) at the end of 2012, followed by an authorisation granted by the European Medicines Agency (EMA) for the use of both DLM and BDQ in adults with MDR-TB in 2013 and 2014, respectively [32].

When deciding which regimen offers the best treatment outcome to the patients, several factors must be considered [31]. The shorter all-oral BDQ-containing regimen is recommended in patients with MDR-TB (with at least confirmed RIF resistance) who fulfill the following eligibility criteria: (1) resistance to fluoroquinolones has been excluded as fluoroquinolones susceptibility testing needs to be undertaken before the start of the shorter regimen, (2) no second-line treatment medicine has been previously administered for more than one month (unless tests were performed to confirm susceptibility to these drugs), (3) no resistance or suspected inefficacy of any drug in the shorter regimen except INH, (4) no severe extrapulmonary disease, (5) no extensive TB disease, (6) no pregnancy and (7) age 6 years and above [31]. If the patient is ineligible for the shorter all-oral regimen or if treatment has to start immediately while drug susceptibility has not been confirmed yet, patient reassessment for a longer all-oral regimen is necessitated [31].

On the other hand, extensively drug-resistant TB (XDR-TB) is a subset of MDR-TB (resistant to INH and RIF) with an additional resistance to at least one fluoroquinolone (such as moxifloxacin or levofloxacin) and any of the injectable second-line TB drugs (such as amikacin) [34]. Therefore, very limited treatment options are available for XDR-TB, resulting in extremely high mortality rates and raising the danger of a return to the pre-antibiotic era [35]. An intermediate stage between MDR-TB and XDR-TB is called pre-XDR-TB, which is an MDR-TB additionally resistant to either a fluoroquinolone or an injectable second-line agent [34]. Pre-XDR-TB and XDR-TB treatment duration ranges between 14–24 months including also both intensive and continuation phases with a combination of the second line agents to which the M. tb strain is susceptible [34]. Recently, a shorter new regimen, comprising BDQ, pretomanid (PMD) and linezolid (BPaL regimen), was approved by the US FDA to be administered in patients with XDR-TB [30]. However, this regimen should only be administered under operational research conditions and when BDQ and linezolid have not been previously used [30]. PMD, which is a nitroimidazole derivative similar to DLM, was the third and most recently approved drug to be added to the TB treatment arsenal [36]. The US FDA granted its approval for PMD in 2019 as a part of the BPaL regimen and limited its indication to adults with XDR-TB or non-responsive or drug-intolerant MDR-TB [36].

People may get MDR-TB or XDR-TB in one of two ways: (1) a primary infection with MDR or XDR bacteria (person-to-person transmission) may occur, or (2) resistance may develop when anti-TB drugs are misused or mismanaged in TB patients [29,30]. Overall, MDR-TB and XDR-TB generally require substantially longer duration of treatment (up to two years) compared to the first-line regimen for DS-TB. Moreover, the second-line anti-TB drugs, recommended for MDR- and XDR-TB, are generally more toxic, more expensive and less efficacious than the front-line drugs [37]. All of which exacerbate the patient adherence dilemma and the spread of the disease in the community, perpetuating TB as a global health menace.

### 4.2. TB and HIV Co-Infection

HIV infection is considered the main predisposing risk factor for patients falling ill with *M. tb*, increasing the likelihood of disease progression into the active stage by 18-fold [4]. Similarly, TB is known to exacerbate the HIV infection and is considered the leading cause of death in HIV patients [38]. In co-infected individuals, both pathogens have profound effects on the immune system, disarming the host’s immune responses and accelerating the decline of the immunological functions [38]. One of the complications of TB and HIV co-infection is devising an appropriate treatment that is attributed to the increased pill burden, overlapping toxic side effects and drug–drug interactions [35]. The main interactions between TB and HIV antibiotics are correlated to RIF-induced elevated expression of hepatic cytochrome P450 (CYP) system. This induction of the CYP enzymes increases the metabolism of several HIV co-medications, such as protease inhibitors, and accordingly decreases their therapeutic concentrations [35]. Even the co-administration of CYP inhibitors, such as ritonavir, cannot salvage the normal trough levels of many different protease inhibitors. Hence, whether boosted or not, standard protease inhibitors cannot be prescribed with RIF [35]. Other rifamycin antibiotic with reduced CYP induction properties is rifabutin, which was identified as an alternative to RIF. However, the co-administration of ritonavir increases the serum concentration and, accordingly, the accompanying toxicity of rifabutin [35]. Taken together, these complications further worsen the clinical management of both infections and the patient adherence to treatment.

### 4.3. The Coronavirus 2019 (COVID-19) Pandemic and TB

TB has long been the world’s leading cause of death from a single infectious disease (surpassing HIV/AIDS since 2007) until the COVID-19 pandemic [4]. Indeed, according to the WHO, COVID-19 caused the deaths of more than 6.7 million people worldwide so far since the start of the pandemic [39]. Before the death figures of TB were released, the WHO predicted that COVID-19 health crisis will have catastrophic effects on the TB deaths rates [4]. Indeed, the WHO indicated that, in 2020 and 2021, an increase in TB deaths was seen for the first time in more than a decade, reversing years of progress made up to 2019 [2,40]. This set-back in the TB control efforts has been attributed to inadequate TB diagnosis and treatment in which nearly half of the patients with active TB were not reported and did not receive care. In addition, alarmingly, there was a significant decline in the provision of TB preventative therapy and DR-TB treatment [2,40].

The substantial decrease, both in 2020 and 2021, in the documented number of people newly diagnosed with TB, indicate that TB transmission has increased in the community due to the grown number of people with untreated and undiagnosed TB. Hence, expectedly, the number of people who died from TB in 2021 (approximately 1.6 million) were higher than the 2020 figures (around 1.5 million) [2,40]. The estimated surge in TB deaths globally was mostly located in four countries, namely, India, Indonesia, the Philippines and Myanmar [2]. The WHO is expecting TB to regain the lead as the deadliest single infectious disease in the near future, replacing COVID-19, which means that the global TB targets have been thrown off track [2]. Indeed, several initiatives, such as Stop TB Partnership, were launched globally with the goal of controlling the TB pandemic. In 2014, the WHO adopted the End TB Strategy (2016–2035), which is aimed at 90% reduction in TB incidents and 95% reduction in TB deaths by 2035 [2]. However, judging by the slow decrease in TB incidence and fatalities in the past two decades in addition to the unfolding crisis of the COVID-19 pandemic, a significant breakthrough is required promptly to accomplish the WHO End TB Strategy objectives.

## 5. TB Drug Targets

### 5.1. Overview

**Figure 5:**
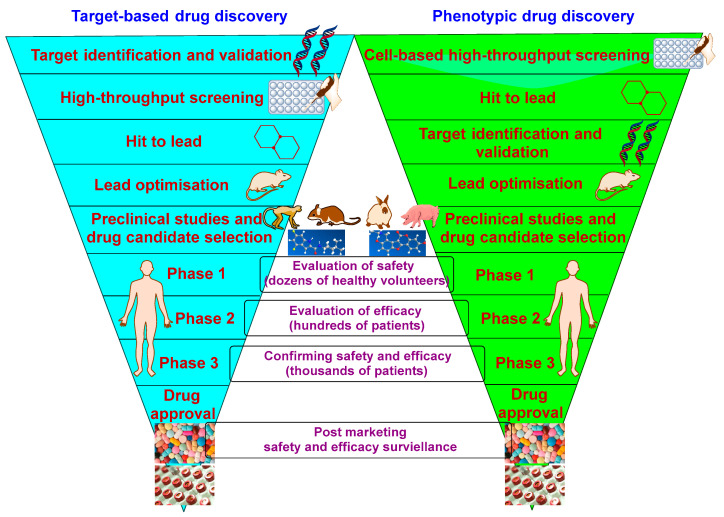
A simplified diagram of target-based and phenotypic TB drug discovery cascade.

In 1998, the complete genome sequencing of *M. tb* (approximately 4000 genes) was unveiled, which advanced our understanding of the molecular biology of the bacterium [41]. Knowledge of the whole-genome *M. tb* sequence enabled researchers to identify a subset of genes that are essential in vitro and in vivo [42]. This revelation in turn contributed to the discovery of new targets for novel compounds via identifying the mutated genes of the strains resistant to these compounds. The gene knockdown techniques, whereby the gene of a specific target is depleted, has also facilitated the validation process of several *M. tb* drug targets [42]. The TB drug discovery approaches can be classified into target-based and phenotypic screening [43,44] (Figure 5). The genome-derived target-based approach (target-to-drug) involves the identification of a specific cellular target in advance but without giving any information about its druggability (drug penetration or efflux) [43]. Indeed, it has been a difficult conundrum to translate a good bacterial enzyme inhibition into a potent whole-cell *M. tb* inhibitory activity because of the difficulty to penetrate the highly impermeable waxy cell wall of *M. tb* [35]. In addition, several inhibitors, which were identified against essential targets, were lacking drug-like properties. Therefore, no anti-TB drug has emerged from this strategy to date [35,43,44].

On the other hand, the cell-based phenotypic screening approach (drug-to-target) is based on a high-throughput screening of compound libraries which proved to be a much more successful strategy [35,43]. In fact, all currently used anti-TB antibiotics were discovered using the phenotypic screening tactic [44]. This approach ensures the compounds’ capability to inhibit bacterial growth at first, followed by identifying their potential target [35,43]. However, the lack of that upfront knowledge regarding the mechanism of action prevents any structural biology input into the drug design efforts by medicinal chemists [35]. Another drawback of the whole-cell screening approach is that, although many hits can be delivered, some of them may have detergent effects; they may work through non-specific mechanisms, thereby having toxic effects. Therefore, to circumvent this problem, the cytotoxicity of the hit compounds should be evaluated across several cell lines to obtain ʺquality hitsʺ with good selectivity and specificity [35]. Overall, the identification of targets for compounds with established anti-TB activity (cell-based/phenotypic approach) allows for the rational modification and optimisation via medicinal chemistry of the lead candidates [43]. Accordingly, adopting this strategy will ensure that the designed compounds retain activity against their primary target.

### 5.2. Current Hot Targets in M. tb Drug Discovery and Their Corresponding TB Drug Candidates

#### 5.2.1. GyrA/B

**Figure 6:**
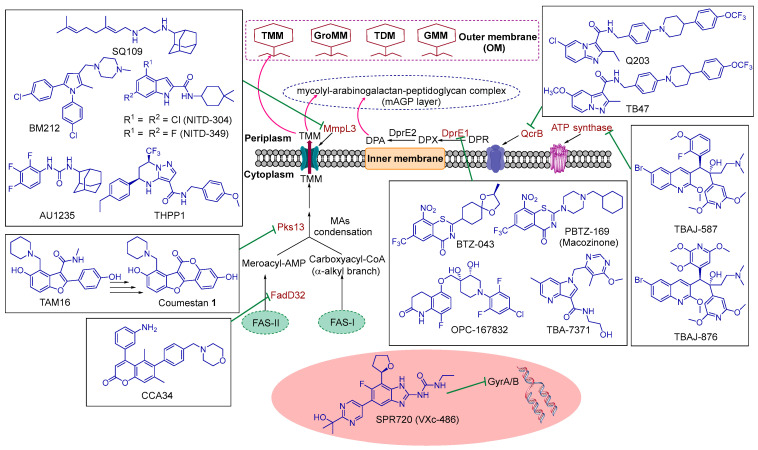
Schematic representation of the site of action of several current promising anti-TB drug candidates and hit/lead compounds. A simple version of the mycobacterial cell wall and the cytoplasmic membrane is portrayed, showing the current hot targets in TB drug discovery, namely GyrA/B, QcrB, ATP synthase, DprE1, FadD32, Pks13 and MmpL3. TMM: trehalose monomycolate, GroMM: glycerol monomycolate, TDM: trehalose dimycolate, GMM: glucose monomycolate (GMM), DPR: decaprenylphosphoryl-D-ribose, DPX: decaprenylphosphoryl-2′-ketoribose, DPA: decaprenylphosphoryl-D-arabinose, MAs: mycolic acids, FAS-I: fatty acid synthase I and FAS-II: fatty acid synthase II.

DNA gyrase is a highly conserved type II topoisomerase enzyme that is essential for DNA transcription, replication and recombination in *M. tb* [45,46]. Therefore, inhibiting DNA gyrase results in impaired DNA replication and permanent double strands breaks, which leads to cytotoxic accumulation of cleaved double-strand DNA fragments, inducing bacterial death [11,47]. DNA gyrase is an ATP-dependent tetrameric enzyme (with A_2_B_2_ heterotetramers), consisting of GyrA and GyrB subunits [45,46]. The GyrA subunit carries the breakage-reunion active site and is a clinically validated drug target of the fluoroquinolone family of antibiotics, such as moxifloxacin. On the other hand, the GyrB subunit (ATPase) promotes ATP hydrolysis and has been relatively less exploited, thereby representing a new avenue for tackling *M. tb* strains that are resistant to fluoroquinolones [45,46]. Indeed, various chemical entities have been developed as GyrB inhibitors, showing potent activity against DR-TB [45]. In particular, a novel class of aminobenzimidazoles was found to target the ATPase subunit, which upon further optimisation led to the discovery of SPR720 (VXc-486) [45] (Figure 6).

SPR720 was found to inhibit a panel of DS and DR *M. tb* isolates in vitro with minimum inhibitory concentrations (MIC) of 0.03–0.30 µg/mL and 0.08–5.48 µg/mL, respectively [45]. It also reduced the *M. tb* burden in the lungs of infected mice in vivo and demonstrated bactericidal activity against intracellular and dormant *M. tb* in a low oxygen environment. Interestingly, the phosphate prodrug of SPR720 showed more potent killing of *M. tb* than the parent compound in vivo. When combined with other anti-TB drugs, the prodrug sterilised the *M. tb* infection in mice with relapse infection [45]. Based on the preclinical efficacy studies of SPR720 in vitro and in vivo against some important non-tuberculous mycobacterial (NTM) species in addition to the toxicology/safety reports obtained thereof, SPR720 (Fobrepodacin) was advanced into human clinical trials [48,49,50]. Similar to *M. tb*, NTM infections can cause progressive lung disease, especially in patients with structural lung damage or weakened immune systems [51]. Phase I clinical trials of SPR720 were initiated in January 2019, aimed at evaluating its tolerability, safety and pharmacokinetics (PK) in healthy volunteers [52].

Towards the end of February 2019, SPR720 was designated the Qualified Infectious Disease Product (QIDP) status by the US FDA for the treatment of lung infections caused by *M. tb* and NTM [53]. In December 2020, Phase IIa clinical trial of SPR720 started on patients with NTM pulmonary disease caused by *Mycobacterium avium* (*M. avium*) complex (MAC). Shortly afterwards, a clinical hold has been placed on SPR720 by the US FDA following concerning events correlated with ongoing animal toxicology studies, wherein mortalities in non-human primates were observed, albeit with inconclusive causality to SPR720 [54]. It is worth noting that there are no specifically approved oral antibiotics for the treatment of pulmonary NTM. Indeed, a prolonged combination therapy with mainly unapproved drugs is recommended (12–24 months) and is often complicated by tolerability and/or safety concerns. Therefore, pending trial results, SPR720 could become the first approved oral antibiotic for NTM infections, addressing a crucial unmet need for the treatment of the debilitating pulmonary disease associated therewith [54].

#### 5.2.2. ATP Synthase

The diarylquinoline BDQ (Figure 4), the most recently approved anti-TB drug with a novel mechanism of action, was found to elicit its activity via inhibiting the *c* subunit of the mycobacterial ATP synthase enzyme [55]. Accordingly, it disrupts the energy metabolism and decreases intracellular ATP levels in *M. tb* [55,56]. However, some issues were associated with BDQ. First, it has an extremely long in vivo elimination half-life and extensive tissue accumulation that could be ascribed to its very high lipophilicity (ClogP = 7.25) [57]. It also showed potent inhibition of the human *ether-a-go-go* gene (hERG) cardiac potassium channel (IC_50_ = 1.6 μM), which is crucial for the repolarisation of cardiac action potentials. This dysfunction of hERG causes prolonged QT (the time interval between the beginning of the Q wave till the end of the T wave) syndrome, resulting in irregular heart rhythm and potentially sudden death [57]. Indeed, BDQ comes with a black box warning for increased risk of arrhythmia and mortality [58]. Therefore, next-generation lead optimisation efforts were subsequently initiated, aimed at lowering the lipophilicity and cardiotoxicity of BDQ and improving clearance while maintaining its high anti-TB activity [57]. In this respect, two diarylquinolines TBAJ-587 and TBAJ-876 were identified (Figure 6). Both compounds have anti-TB activity (MIC_90_ = 0.006 and 0.004 µM, respectively) superior to BDQ (MIC_90_ = 0.03 µM) against H37Rv strain in vitro [57]. In animal models, TBAJ-587 has better efficacy than BDQ while the activity of TBAJ-876 was comparable to BDQ. Importantly, the lipophilicities (ClogP = 5.80 and 5.15, respectively) and hERG inhibitory activities (IC_50_ = 13 and > 30 μM, respectively) of both compounds are lower than BDQ [57]. TBAJ-587 and TBAJ-876 are currently in Phase I clinical trials [48,49].

#### 5.2.3. QcrB

The cytochrome b subunit (QcrB) of the cytochrome *bc_1_* complex has recently emerged as an interesting target in *M. tb* [59]. The cytochrome *bc_1_* complex is a key component of the respiratory electron transport chain required for ATP synthesis. Therefore, the inhibition of this complex disrupts the *M. tb* ability to generate energy. A phenotypic screening of a library encompassing more than 100,000 compounds as antimycobacterial agents led to the identification of imidazopyridine amides (IPAs) as a promising class that blocks the *M. tb* growth by targeting QcrB [59]. An optimised IPA derivative Q203 (Figure 6) showed potent growth inhibition against DS *M. tb* H37Rv strain (MIC_50_ = 2.7 nM) and numerous MDR and XDR *M. tb* clinical isolates in vitro (MIC_90_ < 0.43 nM for most DR strains) [59]. Q203 was found to trigger a rapid ATP depletion in *M. tb* under both aerobic and anaerobic conditions and when a whole-genome sequencing of resistant mutants was conducted, QcrB was identified as its target. Q203 showed minimal cytotoxicity in different eukaryotic cell lines and was well tolerated in mice when administered as a high single dose (1000 mg/kg) as well as in a long-term administration study in rats (10 mg/kg administered for 20 days) [59]. Importantly, Q203 did not inhibit the hERG channel (IC_50_ > 30 µM), suggesting its potential low risk of cardiotoxicity. It also did not induce the human pregnane X receptor (hPXR) at 10 µM concentration and did not inhibit any of the CYP450 isoenzymes tested (IC_50_ > 10 µM). Q203 was also efficacious at a dose < 1 mg/kg in a mouse model of TB [59]. The aforementioned potent anti-TB activities in addition to the promising safety, and PK profiles obtained for Q203 [59] led to its advancement into human clinical trials. Telacebec (Q203) is currently in Phase II clinical trials as an oral antibiotic for treatment of TB. The preliminary results of Phase IIa early bactericidal activity (EBA) demonstrated that Telacebec was well tolerated and safe when administered at different doses to adult patients with pulmonary TB [48,49].

An analogous pyrazolo [1,5-*a*]pyridine-3-carboxamide derivative TB47 (Figure 6) was also identified as a preclinical anti-TB candidate that inhibits QcrB [48,49,60]. TB47 exhibited potent anti-TB activities (MIC = 0.016–0.500 µg/mL) against a panel of *M. tb* clinical isolates, including various MDR and XDR strains [60]. TB47 also showed promising PK and toxicity profiles, whereby it displayed negligible cytotoxicity (IC_50_ > 100 µM against both Vero and HepG2 cell lines), CYP450 interactions (IC_50_ > 20 µM) and hERG channel inhibition (IC_50_ > 30 µM) [60]. In mouse infection models, although TB47 was not bactericidal as a monotherapy, it displayed a strong synergism with PZA and RIF, indicating its potential when combined with other anti-TB drugs [60].

#### 5.2.4. DprE1

Decaprenylphosphoryl-β-D-ribose 2′-epimerase 1 (DprE1), also called decaprenylphosphoryl-β-D-ribose oxidase, is a key enzyme implicated in the mycobacterial cell wall biosynthesis [61,62]. In 2009, a ground-breaking report identified DprE1 as the target of a novel class of inhibitors, namely 1,3-benzothiazin-4-ones (BTZs), that were discovered in a phenotypic screening of a drug library [62]. This new class of compounds is endowed with potent antimycobacterial activities, demonstrating bactericidal activities against *M. tb* in the nanomolar range [62]. DprE1 is a flavoprotein that works in concert with decaprenylphosphoryl-D-2-keto erythro pentose reductase (DprE2) to generate an arabinose precursor that plays a fundamental role in the synthesis of the mycobacterial cell wall polysaccharides arabinogalactan and lipoarabinomannan [61,62]. In this respect, DprE1 uses flavin adenine dinucleotide (FAD) to oxidise decaprenylphosphoryl-D-ribose (DPR) to a keto intermediate [decaprenylphosphoryl-2′-ketoribose (DPX)], which is subsequently reduced by DprE2 to form decaprenylphosphoryl-D-arabinose (DPA) [63] (Figure 6). DPA then serves as a sugar donor for the biogenesis of cell wall arabinans. The DPA biosynthesis was recently shown to take place in the periplasmic space of the mycobacterial cell wall, where DprE1 was also found to be located [63]. The extracytoplasmic localisation of DprE1 makes it more accessible to drugs that contribute to its vulnerability. It was demonstrated that inhibiting DprE1 abolishes the formation of DPA, thereby provoking cell lysis and mycobacterial death [62]. The validity of DprE1 as a drug target was further verified by genetic studies conducted using *M. tb* conditional knock-down mutants [64]. Indeed, the downregulation of DprE1 therein led to bacterial cell wall damage and lysis. Furthermore, rapid death was manifested in the DprE1-depleted mutants in vitro and intracellularly, accentuating its crucial role in bacterial growth and survival [64]. In addition to BTZs, several new classes of DprE1 inhibitors effective against *M. tb* have been identified. Four compounds are currently in advanced stages of clinical trials, namely BTZ-043, PBTZ-169 (Macozinone), OPC-167832 and TBA-7371 (Figure 6).

The benzothiazinone analogue BTZ-043, which is the first identified DprE1 inhibitor, stood out as an exemplar of the BTZs class [62]. In fact, BTZ-043 was found to be a suicide inhibitor of the mycobacterial FAD-dependent DprE1 enzyme, irreversibly inactivating the enzyme by forming a covalent adduct [65]. BTZ-043 displayed nanomolar bactericidal activities both in vitro and ex vivo against *M. tb* [62]. Indeed, BTZ-043 exhibited an MIC value of 1 ng/mL (2.3 nM) against DS H37Rv *M. tb* strain and similar activities against a panel of clinical isolates of *M. tb*, including MDR-TB and XDR-TB strains. In the ex vivo model, BTZ-043 killed *M. tb* intracellularly (MIC < 10 ng/mL) in *M. tb* infected macrophages, demonstrating higher potency than INH and RIF against intracellular bacteria [62]. In murine infection models of TB, the efficacy of BTZ-043 was comparable to INH and RIF, although the in vitro anti-TB activities of the preceding two front-line drugs were far less than that of BTZ-043 [62,66]. In preclinical toxicology studies, BTZ-043 was well tolerated in minipigs (at 360 mg/kg) and rats (up to 170 mg/kg), showing low toxicological potential [48,49]. BTZ-043 showed limited cytotoxic activities against human cell lines, including monocytic THP-1 cells, two hepatic cells (Huh7 and HepG2) and lung epithelial A549 cells [median toxic doses (TD_50_) = 16–77 µg/mL; selectivity indices (SI) = 16,000–77,000] [67]. Phase IIa EBA clinical trials of BTZ-043 commenced in November 2020 [48,49].

Since the exceptional in vitro potency of BTZ-043 did not translate to comparably high in vivo efficacy in TB mouse models, further optimisations were conducted, which led to the development of a new series of enhanced BTZs, namely piperazinebenzothiazinones (PBTZs) [66]. This study was aimed at improving the pharmacological properties of the first-generation lead compound BTZ-043, particularly water solubility, which was achieved by incorporating a piperazine group into the BTZ scaffold. Indeed, the next generation PBTZ-169 (Figure 6) displayed superior physicochemical properties and antimycobacterial activity compared to BTZ-043 [66]. PBTZ-169 (Macozinone) inhibited DprE1 by forming a covalent bond with the cysteine residue in the active site thereof, demonstrating a mechanism of action identical to BTZ-043. The in vitro activity of PBTZ-169 against *M. tb* H37Rv strain (MIC = 0.3 ng/mL) was nearly 3-fold higher than BTZ-043 [66]. Importantly, PBTZ-169 retained its potent activity against a panel of MDR and XDR *M. tb* clinical isolates. Besides its improved in vitro anti-TB potency, PBTZ-169 showed greater promise than BTZ-043 in the following aspects: (1) the lack of chiral centres in PBTZ-169 made the synthesis, manufacture and quality control thereof more convenient than BTZ-043, which decreases its production cost; (2) PBTZ-169 was significantly more efficacious than BTZ-043 in murine models of TB, which may stem from the fact that PBTZ-169 is a more efficient DprE1 inhibitor than BTZ-043; (3) PBTZ-169 displayed less cytotoxicity than BTZ-043; (4) PBTZ-169 demonstrated better solubility than BTZ-043, which accounted for its rapid absorption compared to BTZ-043, indicating a better PK profile [66]. PBTZ-169 acted synergistically with BDQ while additive effects were observed when combined with other anti-TB drugs [66]. Although PBTZ-169 was developed years after BTZ-043 [62,66], PBTZ-169 made a remarkable progress in the clinical trials; therefore, it is currently on par with BTZ043 as both candidates are in Phase II [48,49]. Phase I studies of PBTZ-169 in healthy male volunteers revealed its favourable safety profile and good tolerability. Phase IIa EBA study of PBTZ-169 was completed early 2018, which established its acceptable safety in DS-TB patients. In addition, a statistically significant bactericidal activity was manifested when PBTZ-169 was administered as a monotherapy in a group of seven patients for 14 days [48,49].

Another phenotypic screening campaign conducted on a library of carbostyrils has identified and optimised 3,4-dihydrocarbostyril derivatives with potent anti-TB activities [68]. Notably, the carbostyril structural core constitutes the backbone of numerous drugs and has been recognised for having favourable PK and safety profiles. These efforts led to the identification of the promising anti-TB drug candidate OPC-167832 (Figure 6). Whole genome sequencing of OPC-167832 resistant mutants subsequently identified DprE1 as the target of this compound, and further studies also demonstrated its inhibition of DprE1 enzymatic activity [68]. The MIC values of OPC-167832 against various DS and DR *M. tb* strains ranged from 0.24–2 ng/mL. It showed bactericidal activity against both growing and intracellular *M. tb* at concentrations of 0.5 and 4 ng/mL, respectively. Of note, the killing activity of OPC-167832 against growing *M. tb* was superior to BDQ and linezolid whilst being similar to RIF, moxifloxacin and levofloxacin [68]. This potent bactericidal activity was recapitulated in vivo in a mouse model of chronic TB, in which OPC-167832 displayed a very low minimum effective dose (MED = 0.625 mg/kg). OPC-167832 was also evaluated in combination therapies in TB infected mice, in which it was used alongside DLM as the core component of drug-combination regimens comprising 3 or 4 drugs, whereupon the third and/or fourth drug was linezolid, moxifloxacin or BDQ [68]. The observed sterilising activities of five out of six of these regimens was greater than the front-line regimen (INH, RIF, PZA and EMB). Indeed, the new combinations demonstrated a rapid decrease in the bacterial burden in mice and relapse-preventing effects superior to the standard treatment cocktail [68]. These key attributes resulted in the entry of OPC-167832 into the clinical pipeline (Phase I/II EBA) [48,49].

A series of 4-azaindoles emerged from a scaffold morphing approach based on the imidazopyridine scaffold, exemplified by Q203 [69]. This new class demonstrated excellent in vitro and in vivo anti-TB activities, with DprE1 being identified as their target. In fact, they were found to be non-covalent inhibitors of DprE1 [69,70]. TBA-7371 (Figure 6) was the highlight of this class that proceeded to clinical development [69,71]. This compound displayed potent anti-TB activities against DS and DR *M. tb* strains in vitro (MIC = 0.4–6.25 µM). TBA-7371 also showed potent bactericidal activity against *M. tb* with minimum bactericidal activity (MBC) value of 0.78–1.56 µM and was active against intracellular *M. tb* [69,71]. TBA-7371 was efficacious in a mouse/rat model of chronic TB infection, significantly reducing the bacterial burden in the lungs of infected animals [71]. TBA-7371 showed minimal inhibition of the hERG channel (IC_50_ > 33 µM), suggesting its low risk of cardiotoxicity [71]. When TBA-7371 was tested against THP1 cells (human monocytic cell line), it demonstrated no inhibition up to 100 µM concentration, indicating its lack of cytotoxicity [69,71]. TBA-7371 did not inhibit any of the CYP450 isoenzymes (IC_50_ > 50 µM), suggesting its low tendency for drug–drug interactions. In general, when tested against a panel of human targets, TBA-7371 showed no major safety liabilities [69,71]. When its PK properties were assessed in rodents, good oral exposure was observed [69]. A Phase IIa was initiated in January 2020 to evaluate EBA, safety and PK of TBA-7371 in pulmonary TB [48,49,72].

#### 5.2.5. FadD32 and Pks13

The fatty acyl-AMP ligase 32 (FAAL32 or FadD32), which is also called fatty acid degradation protein D32, and polyketide synthase 13 (Pks13) are crucial enzymes that act in concert with each other, playing pivotal roles in the biosynthetic machinery of MAs (Figure 6) [73,74]. MAs are the major integral lipid components of the exceptionally fortified waxy cell wall of *M. tb* and the primary mediators of hydrophobicity and impermeability thereof [13]. Briefly, in the *M. tb* cytoplasm, the C_24_–C_26_ α-alkyl branch of the MAs and the C_50_–C_60_ meromycolate chain are generated from the fatty acid synthase I (FAS-I) and fatty acid synthase II (FAS-II) systems, respectively. These two fatty acids chains get activated before the final condensation takes place [13]. FadD32 is an adenylating enzyme that activates and transfers the meromycolyl-AMP (meroacyl-AMP) onto the terminal condensing enzyme Pks13. In other words, FadD32 serves as a linking enzyme connecting the FAS and PKS biosynthetic pathways [75]. Pks13 then catalyses a key Claisen condensation reaction, coupling both of the two loaded fatty acyl chains, the α-alkyl branch and meromycolate chain, to produce α-alkyl ꞵ-ketoacids [73]. The resulting assembled chain attach to trehalose, followed by a final reduction step to form trehalose monomycolate (TMM). The formed TMMs serve as MAs precursors, which then get shuttled from cytoplasm to periplasm via the mycobacterial membrane protein large 3 (MmpL3) [73].

The closely partnered triad of FadD32, Pks13 and MmpL3 implicated in the MAs biosynthesis composes a new territory that has not been fully exploited in *M. tb* (Figure 6). Therefore, they represent promising drug targets for the development of new anti-TB agents that could be used in tackling DR-TB. Both *fadD32* and *pks13* genes are adjacent on the same operon (the *fadD32-pks13-accD4* cluster) [76]. MAs contribute to the intrinsic resistance of *M. tb* and are indispensable to mycobacterial survival, persistence and virulence. Therefore, inhibiting crucial enzymes that are involved in the MAs biosynthesis is considered a viable approach in the TB drug discovery [77]. This notion is substantiated by the currently used drugs that target the MAs biosynthesis, exemplified by the well-established anti-TB drug INH which constitutes the backbone of TB chemotherapy along with RIF. Accordingly, inhibiting FadD32 or Pks13 results in impairment in MAs biosynthesis, compromising the integrity of the *M. tb* outer membrane. Indeed, the deletion of the *fadD32* or *pks13* genes in *Corynebacterium glutamicum* abolished the production of MAs, altering the structure of the cell envelope [78,79,80]. In *Mycobacterium smegmatis* (*M. smegmatis*), both genes were also shown to be essential for the mycobacterial growth and survival [78,79].

In *M. tb*, the depletion of *fadD32* was clearly bactericidal and increased the sensitivity of *fadD32* knockdown strain to several antibiotics [81]. A whole-cell phenotypic screening against *M. tb* led to the identification of a series of diarylcoumarins that inhibit FadD32 [82]. The most potent coumarin analogue CCA34 (Figure 6) showed an MIC value of 0.24 µM against DS H37Rv *M. tb* strain [83]. CCA34 also exhibited potent activity against an *M. tb* isolate with monoresistance to INH (MIC = 0.44 µM). In addition, it demonstrated a potent MBC value of 1.9 µM, which is comparable to that of INH (MBC = 0.5 µM). In macrophages infected with *M. tb*, CCA34 was able to kill the intracellular bacilli whilst it had no effect on the macrophage viability [83]. Importantly, in the mouse TB infection models, CCA34 was nontoxic and well-tolerated [maximum tolerated dose (MTD) = 100 mg/kg]. It also inhibited the bacterial proliferation, demonstrating 30-fold reduction in bacterial numbers in the lungs of infected mice after 8 days. This efficacy was found to be comparable to that observed for INH [83]. These findings established FadD32 as a valuable and validated in vivo druggable target for *M. tb*. Since the 4,6-diaryl-5,7-dimethylcoumarins effectively suppress bacterial replication in vivo via inhibiting FadD32, CCA34 could be considered a promising lead compound that can be subjected to further optimisations.

On the other hand, the Sacchettini group have developed a technique that is based on high throughput screening paired with whole-genome sequencing of resistant mutants and recombineering to validate the functional significance of the mutations [84]. This method led to the identification of several whole-cell active compounds and their targets. One of the scaffolds that was identified therein was a benzofuran derivative, which was found to target Pks13 [84]. Upon conducting further structure-based modifications, the same group then highlighted the benzofuran derivative TAM16 (Figure 6) as a potent anti-TB lead compound (MIC values ≤ 0.42 µM). This compound is also endowed with highly potent in vitro bactericidal activities against the tested DS and DR clinical isolates of *M. tb* [85]. It also exhibited potent in vivo efficacy, equal to that of the front-line anti-TB drug INH, in multiple mouse TB infection models. TAM16 demonstrated excellent drug-like properties and favourable safety and PK profiles [85]. TAM16 is currently in the lead optimisation stage [48,49].

Ensuing structure-activity relationship (SAR) optimisation efforts aided by the Pks13 crystal structure led to the identification of several coumestan analogues as potent anti-TB agents in vitro and in vivo in serum inhibition titration assay (SIT) [86,87]. Pks13 was shown to be the target of these coumestan analogues [86]. Coumestan derivative 1 (Figure 6) was the highlight of these published reports, showing potent activities against several DS- and DR-TB strains (MIC/MBC < 0.008 µg/mL) [86]. This excellent in vitro activity was translated into potent in vivo activity in the mouse SIT assay, displaying higher activity than INH and TAM16 [86]. In 2021, further in vitro and in vivo studies were performed on the coumestan analogue 1 [88]. It showed potent sterilising capacity at a concentration of 0.06 µg/mL (15 times the MIC) in culture. In addition, it demonstrated favourable PK parameters when orally administered in mice (10 mg/kg) with a 19.4% relative bioavailability. Importantly, in mouse infection models, coumestan 1 displayed a dose-dependent activity as a monotherapy. It also showed a synergistic effect when combined with RIF (25 mg/kg of 1 and 10 mg/kg of RIF) in reducing the colony forming unit (CFU) in the mouse lungs after 8 weeks of treatment [88]. Taken together, both the benzofuran derivative TAM16 and the coumestan analogue 1 represent promising preclinical anti-TB drug candidates that may undergo further developments in the future.

#### 5.2.6. MmpL3

After the FadD32 and Pks13 crosstalk takes place, which eventually results in the formation of TMM, these MAs precursors then get flipped from cytoplasm to periplasm via the inner membrane protein MmpL3 [76,89] (Figure 6). The mycolyl portion then get anchored to arabinogalactan, the major cell wall polysaccharide, which is further linked to peptidoglycan. It also gets attached to other TMM molecules, glucose and glycerol [89,90]. This anchoring process leads to the formation of the mycolyl-arabinogalactan-peptidoglycan (mAGP) complex and the outer membrane glycolipids trehalose dimycolate (TDM), glucose monomycolate (GMM) and glycerol monomycolate (GroMM) [89,90] (Figure 6). The fundamental role of MmpL3 in shuttling the MAs across the cytoplasmic membrane and, accordingly, forging the formidable permeability barrier of *M. tb* was verified in different reports [91,92,93,94,95]. In this respect, MmpL3 was found to be essential for the replication and viability of *M. tb* [93]. Indeed, the downregulation of the *mmpL3* gene in *M. tb* was associated with an abrogation in mycobacterial division and rapid cell death [91,93]. Not only did silencing *mmpL3* have a bactericidal effect in vitro, but it also reduced the bacterial load in both acute and chronic mouse lung infection models [93]. All of which established the MmpL3 transporter as a well-validated target in *M. tb*.

Several small molecules with diverse chemical entities, including SQ109 [96], indole-2-carboxamides (I2Cs) [97,98,99], AU1235 [92], BM212 [43] and THPP1 [100], have been identified as potent anti-TB agents, and MmpL3 was shown to be their target. In fact, the results of one study favoured a direct mechanism of inhibition of MmpL3 by the preceding five classes of compounds [101]. In the same report, SQ109, BM212 and AU1235 were additionally found to dissipate the proton motive force (PMF) from which MmpL3 derive its energy (indirect mechanism). In 2019, the crystal structure of MmpL3 came to light, elucidating the binding modes of SQ109, I2Cs and AU1235 within the MmpL3 binding pocket [102]. Upon binding, these derivatives occupied three subsites in the proton transportation channel, disrupting the key Asp–Tyr pairs implicated in proton relay and blocking the PMF for substrate translocation [102]. In general, compounds that are targeting MmpL3 are quite lipophilic, which can undermine the water solubility and bioavailability of this class of inhibitors.

Compound SQ109 (Figure 6) is a 1,2-ethylenediamine that was developed from high-throughput screening of EMB analogues and aimed at identifying an EMB-based drug candidate with a decreased toxicity and an improved anti-TB activity [103]. SQ109 is the most advanced MmpL3 inhibitor in the clinical trials (Phase II) [48,49]. Of note, although SQ109 emerged from a combinatorial library based on EMB, both compounds have different structures, and SQ109 was found to have an entirely new mode of action, which is different form EMB [103]. Indeed, a retrospective evaluation of SQ109-resistant mutants divulged a disruption in the assembly of MAs onto the *M. tb* cell wall, which was ascribed to inhibiting the MmpL3 transporter [96]. SQ109 displayed excellent activity against several DS and DR *M. tb* strains in vitro (MIC ≤ 0.78 µg/mL). It was also efficacious in vivo in mouse TB infection models at a dose of 10 mg/kg, which is way below its MTD in mice (600 mg/kg) [103]. SQ109 displayed bactericidal activity with an MBC value of 0.64 µg/mL. SQ109 killed *M. tb* inside macrophages with activity superior to EMB and equivalent to INH [103]. It also reduced the intracellular *M. tb* load by 99% at its MIC and showed synergistic effects when combined with other anti-TB drugs. In preclinical safety studies in dogs, rats and nonhuman primates, the no observed adverse events level (NOAEL) of SQ109 was 30–40 mg/kg/day, depending on the species. SQ109 was safe and well tolerated in Phase I and the preliminary Phase II clinical studies [103]. In a Phase II study, SQ109 showed promising efficacy and tolerability results when added to the standard treatment regimen for patients with pulmonary MDR-TB [48,49].

The whole-cell phenotypic screening technique has also led to the discovery of more classes of MmpL3 inhibitors. In particular, two I2C analogues, NITD-304 and NITD-349 (Figure 6), were previously highlighted as potent anti-TB preclinical candidates for treating MDR-TB [98]. Li et al. employed a combination of in vitro and whole-cell-based approaches and revealed that both NITD-304 and NITD-349 inhibit the MmpL3 via a direct mechanism [101]. Both lead candidates displayed potent activities against DS and DR *M. tb* clinical isolates (MIC_99_ ≤ 0.08 µM) [98]. They exhibited bactericidal activity against in vitro replicating *M. tb* and intramacrophage *M. tb*, in which NITD-304 displayed a bactericidal activity profile similar to isoniazid (INH), rapidly killing *M. tb* at concentrations greater than 0.2 µM. Both compounds also showed favourable oral PK properties in dogs and rodents [98]. The two advanced lead analogues were also efficacious in treating both acute and chronic *M. tb* infections in murine efficacy models [MED = 37.5 (NITD-304) and 25 (NITD-349) mg/kg]. The in vivo activity of both compounds (100 mg/kg each) was comparable to RIF (10 mg/kg) and better than EMB (100 mg/kg). In these mouse infection models, one month of daily dosing (100 mg/kg) of NITD-304 or NITD-349 was well-tolerated in all animals [98]. Furthermore, in vitro and in vivo safety assessment of both candidates, including exploratory two-week rat toxicology studies, revealed their promising safety margin. Indeed, both compounds showed no cytotoxicity towards mammalian cells (CC_50_ ˃ 20 µM) with a selectivity index > 1000. They also showed no inhibition/toxicity in nearly 40 biochemical assays, including a panel of human G protein-coupled receptors, proteases, phosphodiesterases and ion channels (IC_50_ > 30 µM) [98]. Unlike many anti-TB drugs, for instance, moxifloxacin and BDQ, both NITD-304 and NITD-349 were devoid of the cardiotoxic liability, as neither of them inhibited the hERG channel (IC_50_ > 30 µM). Additionally, both candidates neither inhibited nor stimulated the CYP enzymes at 10 µM concentration, except for the CYP2C9 isoform, which was inhibited by NITD-349 at IC_50_ value of 2.67 µM. They also did not induce hPXR activation at 10 µM concentration. Collectively, these findings suggest the low potential for drug–drug interactions correlated with these two I2C analogues [98]. Both compounds are currently in the lead optimisation phase [48,49].

Three hit compounds, AU1235, BM212 and THPP1 (Figure 6), were also shown to have potent bactericidal activities against *M. tb* via targeting MmpL3 [92,100,104]. The adamantyl urea derivative AU1235 demonstrated potent activities against DS and DR *M. tb* strains (MIC < 0.12 µg/mL) while having negligible cytotoxicity against mammalian Vero cells (IC_50_ = 219 µg/mL) [92,105]. The diarylpyrrole derivative BM212 (Figure 6) exhibited strong inhibitory activities against several *M. tb* strains, including MDR-TB (MIC = 0.7–1.5 µg/mL). It also displayed bactericidal activity against intracellular *M. tb* (MIC = 0.5 µg/mL) with no macrophage loss detected [104]. Finally, the tetrahydropyrazolo [1,5-*a*]pyrimidine-3-carboxamide compound (THPP1) showed potent anti-TB activities against a panel of DS and mono-resistant *M. tb* strains (MIC = 0.16–0.6 µM) in addition to MDR and XDR *M. tb* strains (MIC = 0.16–5 µM) [100]. THPP1 also demonstrated a potent intracellular anti-TB activity with MIC value of 0.16 µM in infected murine macrophages. THPP1 also exhibited minimal cytotoxicity against human HepG2 cells (IC_50_ > 25 µM) [100].

## 6. Concluding Remarks

Despite the availability of potentially curative antibiotics, TB continues to cause morbidity and mortality at alarming rates worldwide, especially in developing countries. The tubercle bacilli are typically constrained by granulomas in immunocompetent individuals, wherein a lifelong standoff between the bacteria and the host’s immune system takes place (latent TB infection). This covert (asymptomatic) TB infection can recrudesce when the host immunity is impaired, which results in a high bacterial burden and the progression of the disease, culminating in clinical manifestations and TB transmission. Treating DS *M. tb* infections is usually attainable with the first-line anti-TB drug regimen. However, managing DR-TB infections is more challenging and less promising, leading to the continued relentlessness of the TB pandemic. In addition, the TB control efforts are generally hampered by the HIV co-infection, COVID-19, poor patient compliance and suboptimal treatment approaches in different parts of the world.

Since the whole genome sequencing of *M. tb* (≈4000 genes) was revealed, a multitude of small molecules with potent activities against both DS and DR *M. tb* strains were discovered, and their targets were identified and validated. Indeed, many scientists have been focusing their research efforts on newly identified *M. tb* drug targets, diverting from the traditional targets of the currently used TB antibiotics to bypass the DR issue. The most prominent drug targets that have recently been attracting attention involve GyrA/B, ATP synthase, QcrB, DprE1, FadD32, Pks13 and MmpL3. Unfortunately, a few of the drug candidates that inhibit some of the preceding targets were found to have toxicity, insufficient in vivo activity or elimination half-life issues. Indeed, despite the extensive efforts undertaken to date to introduce more efficacious anti-TB drugs to the market, only three medications working through new mechanisms were approved since 2013 in more than five decades and are correlated with serious side effects. Therefore, feeding the TB drug development pipeline with new highly active drug-like anti-TB molecules may help expedite the discovery of revolutionary TB medications.

## Data Availability

Not applicable.
